# Suspension button constructs restore posterior knee laxity in solid tibial avulsion of the posterior cruciate ligament

**DOI:** 10.1007/s00167-021-06510-1

**Published:** 2021-03-06

**Authors:** Philipp Forkel, Louis Buchmann, Jan J. Lang, Rainer Burgkart, Andreas B. Imhoff, Julian Mehl, Matthias J. Feucht, Patrizia Lutz, Andreas Schmitt

**Affiliations:** 1 Department of Sports Orthopaedic Medicine, Klinikum Rechts Der Isar, TU Munich, Ismaninger Str. 22, 81675 München, Germany; 2 Department of Orthopaedics and Sport Orthopaedics, Klinikum Rechts Der Isar, TU Munich, Ismaninger Str. 22, 81675 München, Germany; 3 Chair of Non-Destructive Testing, Department of Mechanical Engineering, TU Munich, Franz-Langinger-Straße 10, 81245 München, Germany

**Keywords:** PCL, Tibial avulsion, Posterior knee laxity, Suspension button

## Abstract

**Purpose:**

Dislocated tibial avulsions of the posterior cruciate ligament (PCL) require surgical intervention. Several arthroscopic strategies are options to fix the fragment and restore posterior laxity, including two types of suspension button devices: adjustable (self-locking) and rigid knotted systems. Our hypothesis was that a rigid knotted button construct has superior biomechanical properties regarding laxity restoration compared with an adjustable system. Both techniques were compared with standard screw fixation and the native PCL.

**Methods:**

Sixty porcine knees were dissected. The constructs were tested for elongation, stiffness, yield force, load to failure force, and failure mode in a material testing machine. Group N (native, intact PCL) was used as a control group. In group DB (Dogbone™), TR (Tightrope™), and S (screw), a standardized block osteotomy with the osteotomized fragment attached to the PCL was set. The DB and TR groups simulated using a suspension button system with either a rigid knotted (DB) or adjustable system (TR). These groups were compared to a screw technique (S) simulating antegrade screw fixation from posterior.

**Results:**

Comparing the different techniques (DB, TR, S), no significant elongation was detected; all techniques achieved a sufficient posterior laxity restoration. Significant elongation in the DB and TR group was detected compared with the native PCL (N). In contrast, screw fixation did not lead to significant elongation. The stiffness, yield load, and load to failure force did not differ significantly between the techniques. None of the techniques reached the same level of yield load and load to failure force as the intact state.

**Conclusion:**

Arthroscopic suspension button techniques sufficiently restore the posterior laxity and gain a comparable construct strength as an open antegrade screw fixation.

## Introduction

Tibial avulsion of the posterior cruciate ligament (PCL) is a frequent cause of posterior knee instability, and dislocated fractures require surgical intervention. The type of surgery depends on fragment size and fragment shape, and open reduction and screw fixation are the typical treatments of choice in cases with a solid fracture fragment [15, 22]. With small or comminuted fragments, open reduction can be performed using a hook plate or toothed plate [5, 24], which guarantee solid fragment fixation but require implant removal if revision is necessary. To overcome this disadvantage, open reduction and fragment fixation can be performed using a suture bridge technique [8, 19, 23]. In contrast to open techniques, arthroscopic techniques avoid an open approach and enable the surgeon to treat additional intra-articular injuries in a one-step procedure [6, 10, 11, 23]. Satisfactory results can be achieved using these techniques [10, 13]. Suspension button devices constitute two metal buttons with the bone set between the buttons, and fracture reduction is maintained by a suture or tape between the buttons. Two systems are available: an open adjustable loop system (Tightrope™; Arthrex, Naples, USA) [11] or a rigid knotted loop system (FiberTape™ and Dogbone™; Arthrex) [23].

Adjustable suspension button devices are used to reduce tibial anterior cruciate ligament (ACL) avulsions [1] or tibial PCL avulsions [11]. The advantage of these adjustable systems in contrast to rigid fixation is their feasibility and easy application during surgery. The finger trap closes by pulling on the loose sutures, and fracture reduction is easily secured. Several studies have investigated the biomechanical properties of adjustable and knotted loop systems regarding soft tissue graft fixation in ACL reconstruction [2, 4, 14, 16, 17]. As these constructs can be used to fix PCL avulsion fractures, the aim of our study was to investigate the capability of these suspension button constructs, either adjustable or knotted, to restore posterior knee laxity after osteotomy of the tibial PCL insertion. The fixation strength, elongation, and failure mode of these techniques were measured.

The hypothesis of this study was that fixation of the osteotomized PCL fragment would lead to safe restoration of posterior knee laxity. Additionally, an arthroscopic technique using a rigid suspension button device would be superior in posterior knee laxity restoration compared with the adjustable system. Both techniques were compared to screw fixation and the native PCL.

## Materials and methods

Institutional Review Board (IRB) approval was not required for this study.

Sixty fresh-frozen porcine knee joints from swine 24 weeks of age were used for biomechanical testing. Before dissection, the knees were thawed at room temperature for 24 h, and during dissection, the capsule and the anterior extensor apparatus were resected. The collateral ligaments, ACL, PCL, and the menisci were kept intact. The specimens were embedded in a custom-made three-dimensional (3D)-printed prismatic cast (polylactic acid, 70 × 90 mm^2^, 90 mm in height) using rapid-setting polyurethane resin (RenCast^®^ FC-52/53 Isocyanate/FC52 Polyol; Huntsman Advanced Technology Center, The Woodlands, TX, USA). The specimens were kept moist with a 0.9% sodium chloride (NaCl) solution during the entire test.

### Study groups

**Fig. 1:**
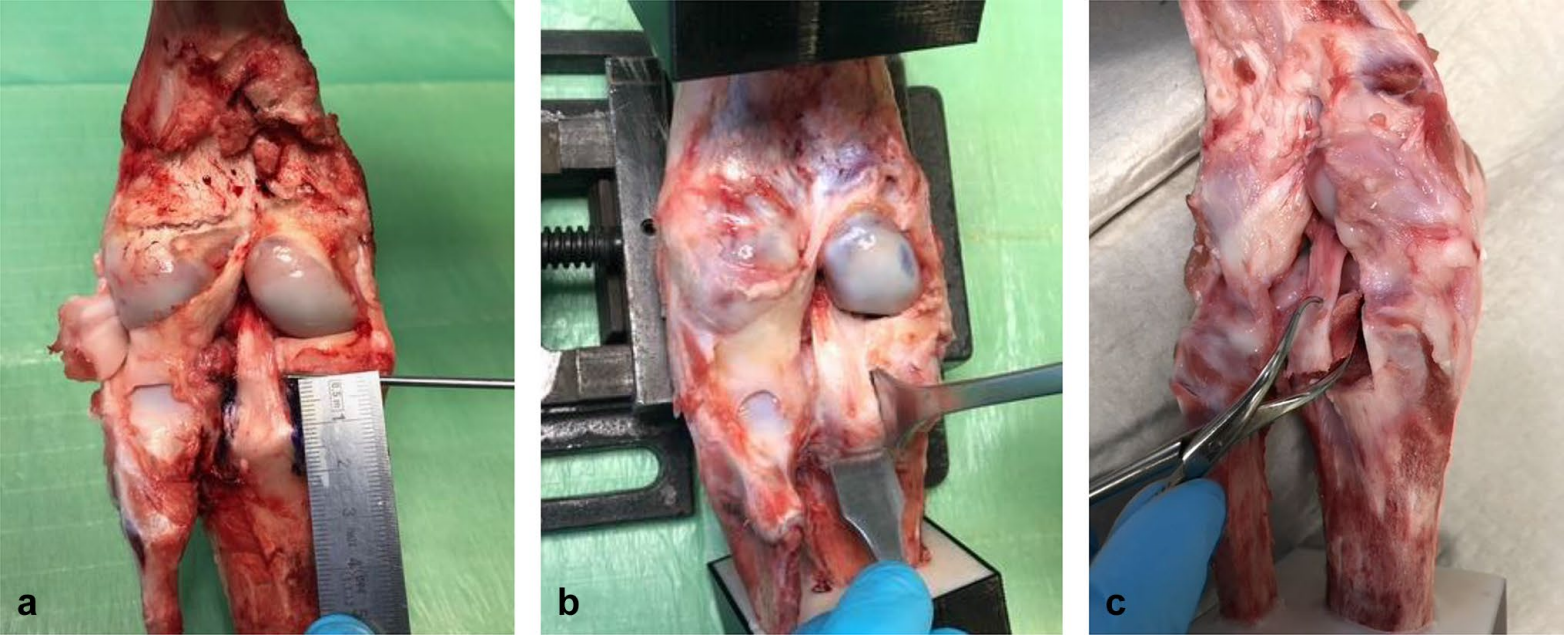
**a** Preparing the PCL attachment model in the porcine knee; (**b**) Standardized osteotomy of the tibial PCL attachment; (**c**) The osteotomized solid fragment is attached to the PCL fibers. *PCL* posterior cruciate ligament

To simulate a solid fracture fragment, a standardized block osteotomy with the osteotomized fragment attached to the tibial insertion of the PCL was created using a chisel. The average fragment size was 20 mm (length) × 15 mm (width) × 10 mm (depth). The knees were randomized into four groups (*N* = 15) to compare the two suspension button devices (Group TR: Tightrope™ plus Dogbone™ and Group DB: Dogbone™ and knotted FiberTape™) with the native PCL (Group N) and solid screw fixation (Group S) (Fig. 1).

**Fig. 2:**
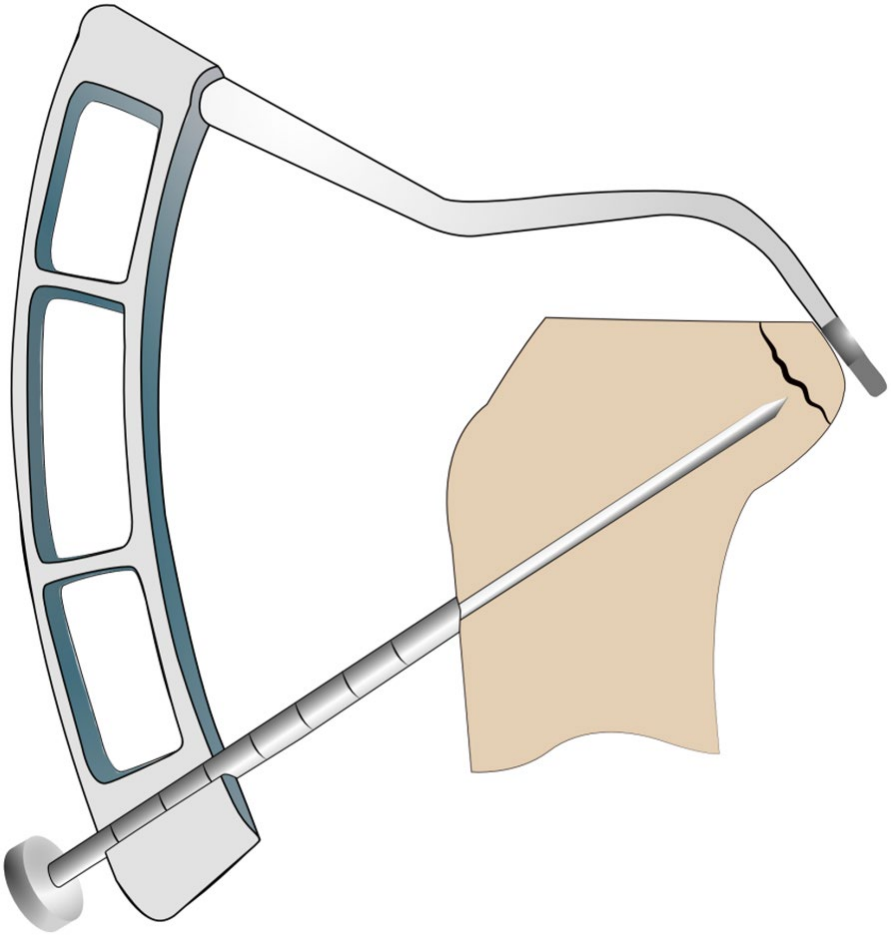
Illustration of the posterior cruciate ligament guiding device used intraoperatively to position the 2.4-mm bone tunnel. Then, either the Fiber tape™ or the Tightrope™ system can be inserted via the 2.4-mm drill hole

**Fig. 3:**
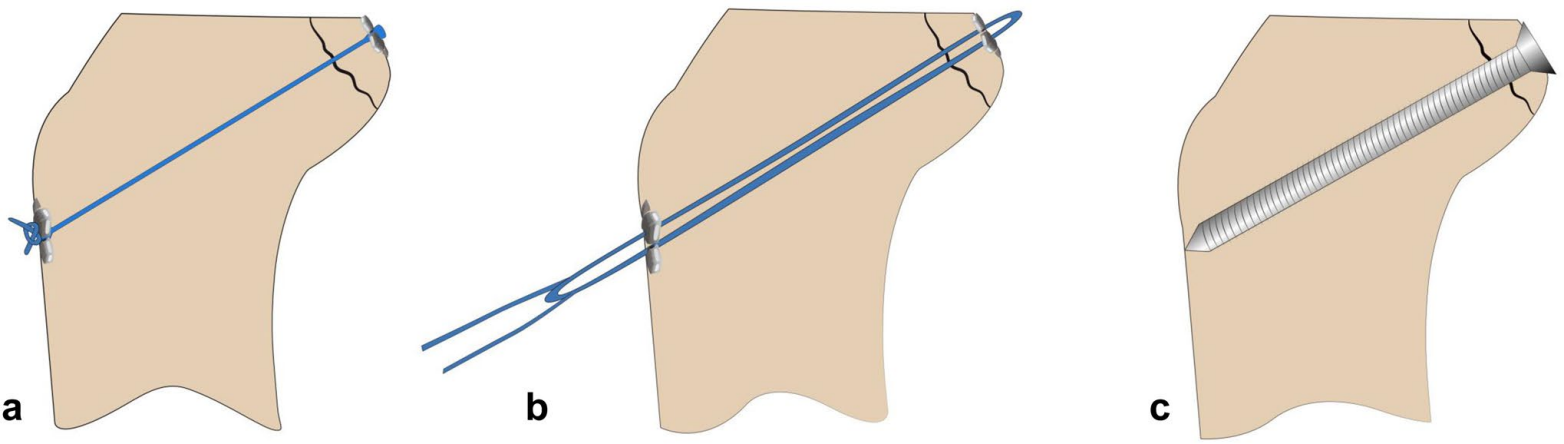
Illustration of the three fixation techniques used in this study. **a** The dogbone technique uses two Dogbone™ devices. The Fiber Tape™ is knotted against the anterior button. **b** The tightrope technique uses two Tightrope™ devices. The self-locking Tightrope™ mechanism acts like a Chinese finger trap by pulling the free sutures. **c** The screw technique uses a solid 4-mm partially-threaded screw from a posterior approach

To simulate the arthroscopic fixation technique for both suspension button constructs, the fragment was reduced in the fracture site, and a 2.4-mm drill hole was created at a 45-degree angle through the avulsion fragment and the tibial head, penetrating the anterior cortex medial to the tibial tuberosity. Next, a lasso loop was brought through the cannulated drill, and either a TightRope™ sling or a FiberTape™ was inserted. Two Dogbone™ buttons were clamped either in the TightRope™ or the Fiber Tape™. The buttons guaranteed a press fit fixation of the avulsion fragment to the fracture site when the loose fibers of the TightRope™ were pulled, which locked the finger-trap mechanism, or when the FiberTape™ was pulled, and the tape was knotted above the anterior button (Figs. 2 and 3).

Screw fixation was performed as for open fixation. An antegrade screw was positioned from posterior in the same direction as when using the suspension button devices, meeting the anterior cortex medial to the tibial tuberosity. A 3.2-mm drill hole was created, and a 4-mm partially-threaded compression screw was used. The length of the screws was adapted in accordance with the length of the drill holes and ranged from 40 to 50 mm (Fig. 3).

### Biomechanical test setup

Biomechanical testing was performed according to two previously published studies [6, 8].

The embedded specimens were placed in an uniaxial hydrodynamic material testing system (Amsler HC 10; Zwick/Roell, Ulm, Germany) in 90° knee flexion. The tibia was secured horizontally, and the femur was vertically mounted unconstrained in the coronal plane of the femur in the testing machine. An axial load was applied on the femur to create a posterior drawer force, and the weight of the femur and the proximal embedding resin were compensated to guarantee force values expressing only the knee loading. A 5-N load on the load cell was determined as the starting position. The knees were pre-conditioned 10 times with a load between 5 and 20 N prior to measurement to minimize the viscoelastic effects. In accordance with previous studies, the specimens were subsequently cyclically loaded 500 times between 10 and 100 N at a frequency of 1 Hz [2, 15]. After cyclic loading, the specimens were loaded to failure at a displacement rate of 200 mm/min. Elongation, initial and final stiffness, yield force, and ultimate load to failure force were calculated from the recorded data. The yield force corresponds to the yield point and describes the strength leading to plastic deformity of a construct. The initial and final stiffness values describe the gradient between the forces of 10 N and 100 N over the associated strains at the 10th and 500th repetitions during the cyclic loading. The 10th repetition for the initial stiffness was chosen because the required adjustment of the machines’ peak value controller was typically completed after the first 3–9 repetitions. Finally, the failure mode was documented.

Owing to a technical error during the testing procedure, TR group had to be excluded by two specimens.

### Statistical analysis

An a priori power analysis was performed on the basis of three groups of three specimens. The groups constituted the native (N), Dogbone^™^ (DB) and Tightrope^™^ (TR) groups. A sample size of 15 specimens provided at least 80% power to detect a significant difference regarding elongation at *α* = 0.05. Measurements were evaluated using Microsoft Excel^©^ and Matlab. Normal distribution of the data was examined and graphically confirmed with the Shapiro–Wilk’s normality test. The data were reported as means and standard deviations. SPSS 21 was used for the statistical calculations, and the four groups were compared regarding elongation, stiffness, load to failure force, and yield load using analysis of variance. A post hoc Bonferroni correction was performed to account for multiple comparisons, and significance was set at *P* < 0.05.

## Results:

### Elongation

**Fig. 4:**
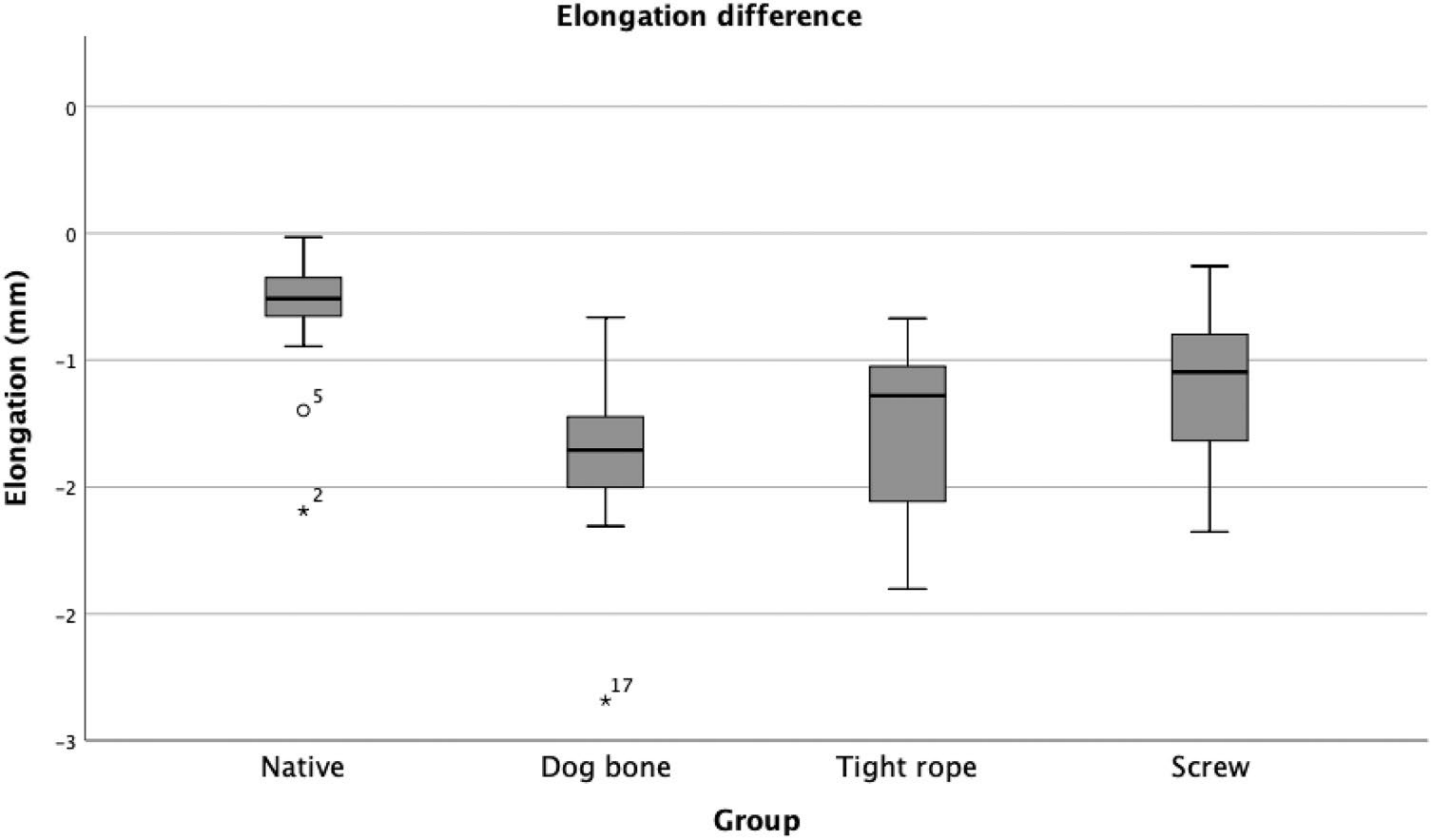
Boxplot Diagram: Elongation (mm). The TR, DB and S groups did not differ significantly. The elongation behavior of the Screw fixation was not significantly increased compared with the intact state. Compared with the native posterior cruciate ligament, the TR and DB groups showed an elongation. *TR* Tightrope^™^; *DB* Dogbone^™^ ;*S* screw

The comparison of the three fixation techniques (DB, TR, and S) revealed no significant difference regarding elongation. However, both suspension button constructs (DB, TR) showed a statistically significant increase in elongation compared with the native PCL during cyclic loading. Screw fixation (S) led to increased elongation but did not reach statistical significance compared with the native PCL (Table 1; Fig. 4).

**Table 1:** Elongation

	*n*	Elongation and SD (mm)	Native *P* value	Dogbone *P* value	Tightrope *P* value
Native	15	0.82 ± 0.27	–	–	–
Dogbone	15	1.39 ± 0.35	< 0.001	–	–
Tightrope	13	1.31 ± 0.37	0.001	n.s	-
Screw	15	1.11 ± 0.29	n.s	n.s	n.s

The elongation values for all groups are reported in mm

### Stiffness

**Fig. 5:**
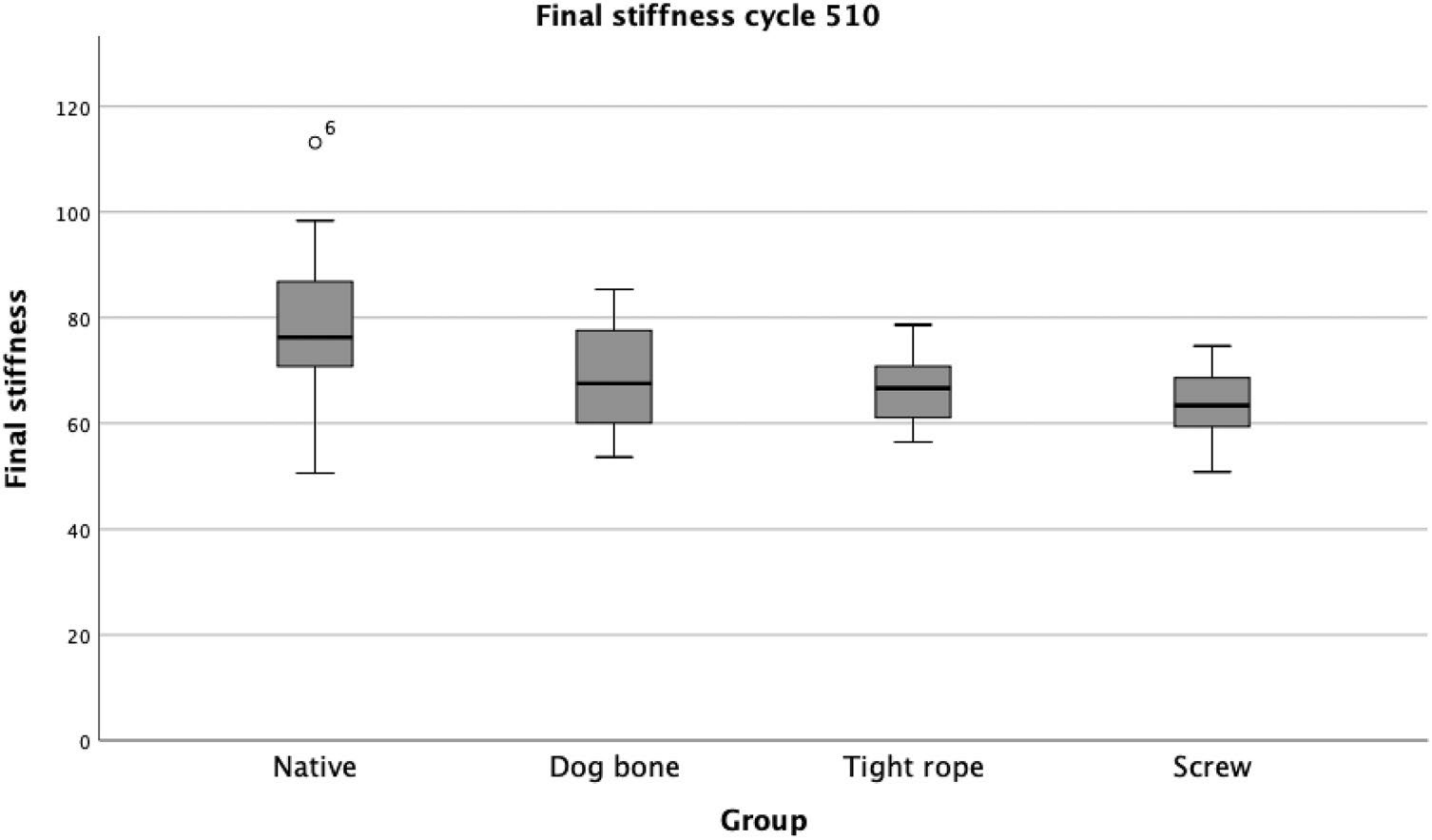
Boxplot Diagram: Final stiffness (N/mm^2^). Compared with the native group, the DB group showed no significant reduction of stiffness; the screw and TR groups showed a decrease. *DB* Dogbone™

Comparing the stiffness (at 510 cycles), only the DB group reached similar values to those with the native PCL. The TR group and the S group had significantly lower stiffness values (Table 2, Fig. 5).

**Table 2:** Final stiffness

	*n*	Stiffness and SD (Nmm^2^)	Native *P* value	Dogbone *P* value	Tightropec *P* value
Native	15	79.14 ± 15.10	–	–	–
Dogbone	15	68.80 ± 10.17	n.s	–	–
Tightrope	13	67.52 ± 7.27	0.019	n.s	–
Screw	15	63.29 ± 6.93	0.001	n.s	n.s

The final stiffnes values for all groups are reported in N/mm^2^

### Yield force

**Fig. 6:**
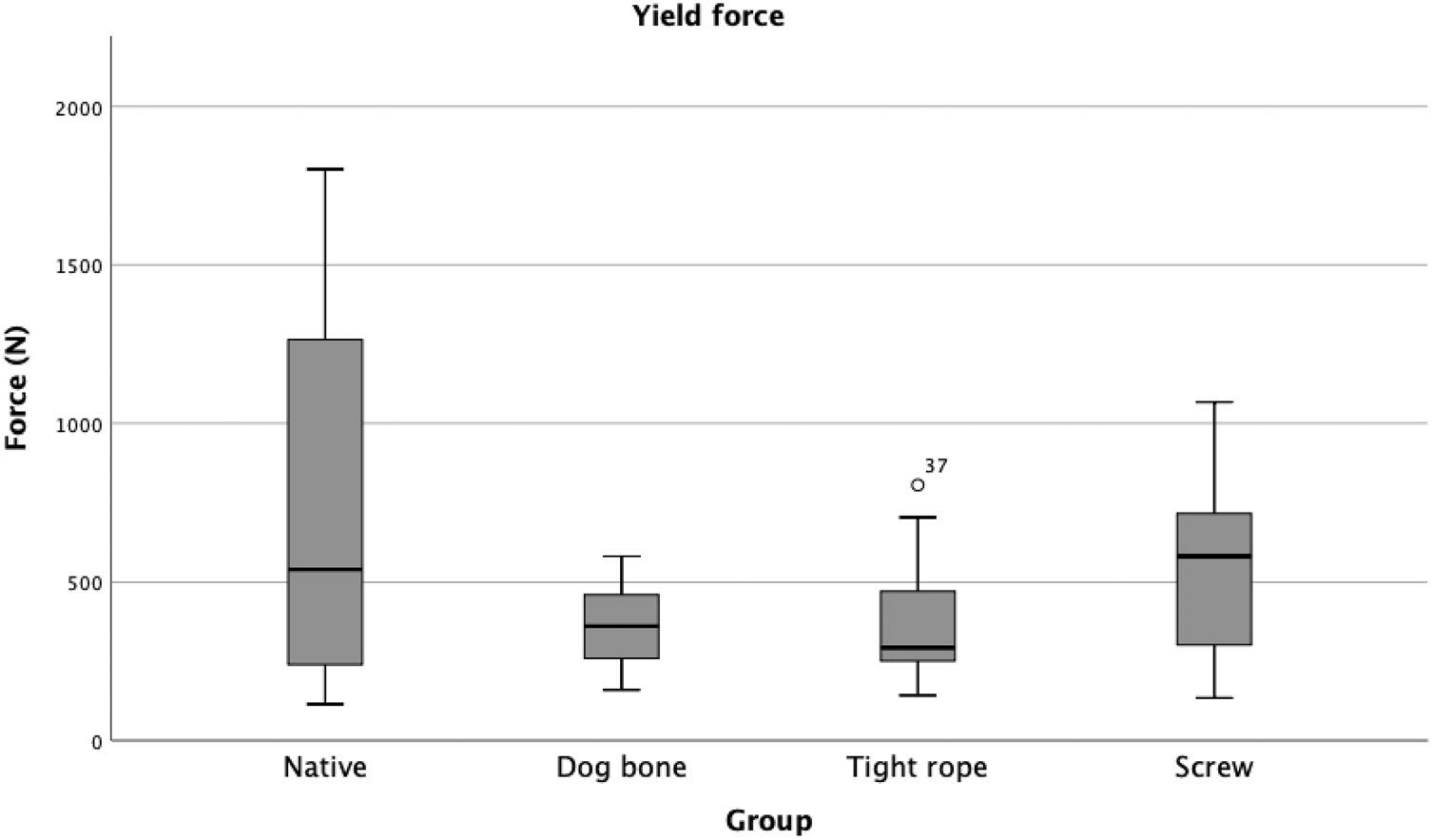
Boxplot Diagram: Yield force (*N*). The yield force was obviously lower in all groups compared with the native posterior cruciate ligament. The yield force was significantly lower only in the DB group. *DB* Dogbone™

The yield force was lower for all three reconstruction techniques compared with the intact PCL; however, the lower yield force in the DB group was significant. The other fixation techniques (S and TR) indicated an obviously lower yield force, but values did not reach statistical significance compared with the native PCL (Table 3; Fig. 6). Comparisons of the three techniques did not reveal a statistically significant difference in yield load values.

**Table 3:** Yield force

	*n*	Yield force and SD (*N*)	Native*P* value	Dogbone *P* value	Tightrope *P* value
Native	15	770.53 ± 638.11	–	–	–
Dogbone	15	361.68 ± 125.15	0.027	–	–
Tightrope	13	383.10 ± 220.13	n.s	n.s	–
Screw	15	532.24 ± 289.86	n.s	n.s	n.s

The yield force is given in *N*

### Maximum force

**Fig. 7:**
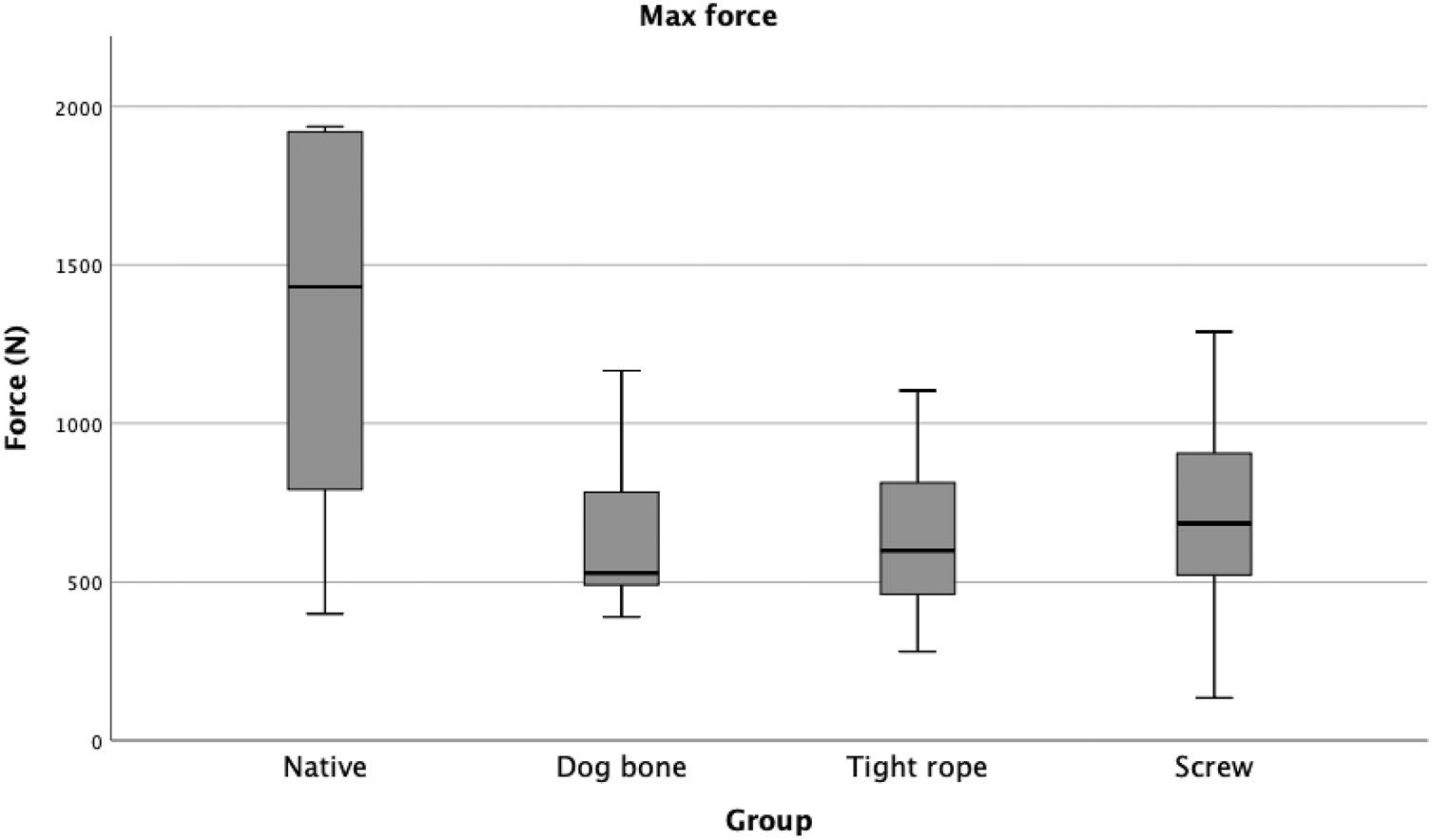
Boxplot Diagram: Maximum Force (*N*). None of the groups (S, TR, DB) reached the maximum force of the native PCL. No significant difference between the reconstruction groups was detected. *S* screw; *TR* Tightrope™; *DB* Dogbone™

No fixation technique reached the maximum force value of the native PCL, and there was no significant difference when comparing the different fixation techniques with each other (Table 4; Fig. 7).

**Table 4:** Maximum force

	*n*	Maximum force and SD (*N*)	Native *p* value	Dogbone *P* value	Tightrope *p* value
Native	15	1326.45 ± 598.39	–	–	–
Dogbone	15	643.73 ± 243.03	0.001	–	–
Tightrope	13	645.93 ± 243.46	0.001	n.s	–
Screw	15	681.54 ± 311.25	0.001	n.s	n.s

The maximum force is presented in *N*

### Failure mode

The failure modes are described in Table 5. The native PCL revealed two typical failure modes; fiber distortion or osseous avulsion of the PCL attachment were noted. The typical failure mode of the DB was a cutout of the construct through the osteotomized bone fragment. In two cases, rupture of the enthesis of the PCL fibers occurred. Interestingly, the TR group revealed a similar failure mode; cutout of the construct was noted in 10 cases. Two specimens showed elongation of the construct during the cyclic loading test (1.8 mm and 1.9 mm, respectively) and ongoing elongation of the construct during the load to failure testing. One specimen failed to elongate, but only during the load to failure test. Screw fixation failed in 10 cases after breakage of the osteotomized bone around the screw, similar to the cut-out failure seen in the TR and DB groups. PCL rupture from its enthesis was noted in five cases.

**Table 5:** Failure mode

	*n*	Failure mode
Native	15	7 × tibial osseus PCL avulsions/8 × Distorsion of the PCL
Dogbone	15	13 × cut out of the construct/2 × avulsion of the enthesis of the PCL
Tightrope	13	11 × cut out of the construct/3 × elongation of the construct
Screw	15	10 × breakage of the fragment around the screw “cut out”/ 5 × avulsion of the enthesis of the PCL

The TR group showed elongation in three specimens.Two specimen elongated during cyclic loading; one specimen elongated during load to failure testing

## Discussion

The most important finding in this study was the capability of the three fixation techniques to restore posterior knee laxity in a standardized osseous PCL osteotomy model simulating an avulsion fracture. Although the three techniques did not restore the biomechanical properties of the native PCL, all techniques led to acceptable values for strength and resistance against posterior drawer in this porcine knee model.

Our initial hypothesis was partially confirmed by our findings. Interestingly, the comparison of the two suspension button techniques did not indicate statistically significant differences. Only the comparison of their failure modes showed elongation of the TR construct in three cases. Under cyclic loading, two specimens began to fail and elongated 1.8 mm and 1.9 mm, respectively. The third specimen elongated during load to failure testing, only. The measured elongation under cyclic loading in this specimen was 0.99 mm. However, both suspension button constructs (DB and TR) were comparable to rigid antegrade screw fixation regarding their elongation and load to failure results.

Adjustable loop systems are frequently used in anterior or posterior cruciate ligament reconstruction and guarantee cortical graft fixation. Biomechanical studies investigating the properties of extracortical soft tissue fixation in the femoral bone tunnel suggest that adjustable devices do not provide equal stability and show higher elongation compared with rigid loop systems [2, 4, 9, 14, 17]. Debate continues regarding whether adjustable loop fixation devices lead to reduced knee stability because of elongation during the healing process in ACL or PCL reconstruction. However, these devices are frequently used clinically, particularly because of their ease of handling. In a recent clinical trial, Kusano et al. found no clinically meaningful differences in postoperative loop length and loop length after 2 years following bone-patellar tendon-bone ACL repair (BTB-ACLR) using the BTB-TightRope™ (Arthrex) and described good subjective results and side-to-side restoration of laxity [18].

The use of suspension button devices to reduce and secure tibial eminence fractures (ACL) or tibial PCL avulsions is a new procedure in arthroscopic knee surgery [1, 10, 11].

Gwinner and Jung reported good clinical results in a preliminary cohort study of osseous PCL avulsions treated with an adjustable suspension button device (TightRope™). The authors confirmed complete osseous integration after a mean follow-up of 22 months [10]. Domnick et al. [6] reported an arthroscopic procedure using a rigid suspension button device in solid tibial PCL avulsion [7]. In their biomechanical study, Domnick et al. found that a fixed suspension button device restored knee stability better than antegrade screw fixation using a posterior approach. The mean elongation was 1.3 mm when a suspension button device was used; antegrade screw fixation led to an elongation of 2.2 mm [6]. Similar values were found in this study for tibial translation with suspension buttons, and with the rigid knotted loop system (DB); the mean elongation was 1.4 mm, while the adjustable loop system (TR) elongated only 1.3 mm. Elongation in the native knee was 0.8 mm, in our study. In contrast to the findings of Domnick et al., antegrade screw fixation provided the lowest elongation values (1.1 mm), in our study. However, the measured values of construct elongation with the fixation techniques were not statistically significantly different.

A previous study investigated the biomechanical properties of suture bridge fixation of smaller and thinner fragments from the PCL insertion. The measured mean elongation of the construct was 4.5 mm, and the load to failure force was only 286 N. The authors concluded that a restricted rehabilitation protocol with nonloading and preventing posterior drawer was necessary until complete bone healing [8]. In contrast, the current study simulated the different fixation options of a larger and solid PCL avulsion fracture. The measured values for the load to failure force, yield force, and stiffness were higher compared with values for the previously reported suture bridge technique.

Our results showed that an adjustable loop system can be used with equivalent stability values compared with a screw or rigid suspension buttons. However, elongation of all three constructs was obvious in this group. Additional knotting of the Tightrope ™ construct might be reasonable, but was not tested in this study.

The applicability of our findings to clinical practice might be difficult to determine, and our findings must be interpreted with caution. However, the porcine knee joint model used in this study was chosen because the knees are a standardized size and bone quality, and the knee structures are generally intact. Nagarkatti et al. found similar values for porcine bone density compared with young human bones, and porcine bone density was even higher compared with elderly human cadaveric bone specimens [20]. Ayzenberg et al. performed femoral ACL anchor fixation and found similar results regarding stability in human and porcine knee joints, indicating equal stability of both simulation procedures (human and porcine) [3]. Porcine knees are used frequently in biomechanical studies. Heitmann et al. tested different fixations and bracing techniques in ACL injury in the porcine knee [12], and Smith et al. tested different techniques and ACL fixation devices [21]. Our model is comparable to those in other studies regarding the setup and the testing protocol (force and cycles) [6, 8, 12].

## Conclusion

Arthroscopic techniques allow for safe posterior laxity restoration compared with open antegrade screw fixation. A posterior open approach to fix these solid fragments can be avoided, and time-consuming intraoperative shifting of the patient’s position or a two-step procedure is unnecessary.
